# Response regarding Colombian regulation of biotherapeutic products

**DOI:** 10.26633/RPSP.2017.129

**Published:** 2017-09-11

**Authors:** Claudia Vaca, Alejandro Gaviria, Carolina Gómez

**Affiliations:** 1 Associate Professor of Pharma-Epidemiology Director of Think Tank “Medicines, Information and Power” Universidad Nacional de Colombia Bogotá Colombia Associate Professor of Pharma-Epidemiology Director of Think Tank “Medicines, Information and Power” Universidad Nacional de Colombia Bogotá, Colombia; 2 Ministry of Health Bogotá Ministry of Health Bogotá Colombia Ministry of Health Bogotá, Colombia; 3 Think Tank “Medicines, Information and Power” Universidad Nacional de Colombia Bogotá Colombia Think Tank “Medicines, Information and Power” Universidad Nacional de Colombia Bogotá, Colombia

Regarding a letter by Erika Lietzan (1) addressing our article on regulating biotechnology drugs in Colombia (2), we offer the following response. As Lietzan states, it is true that there is a global consensus on the two regulatory pathways to obtaining market authorization for biological medicines: the full dossier pathway for pioneer products and an abbreviated pathway for competitors (biosimilars), called the “comparability pathway” in Colombia. The scientific principles of the abbreviated pathway are clearly stated in our article, referencing the *WHO Guidelines on Evaluation of Similar Biotherapeutic Products* (3), which underscores the importance of demonstrating a high similarity of physicochemical and functional characteristics between the biocompetitor and the reference product.

It is also true, as stated by Lietzan, that the United States Food and Drug Administration (FDA) and the European Medicines Agency (EMA) accept that, in specific circumstances, a confir-matory clinical trial may not be necessary when the efficacy and safety can clearly be deduced from the similarity of physicochemical characteristics, biological activity/potency, and pharmacokinetic (PK) and/or pharmacodynamic (PD) profiles of the biosimilar and the reference product. In other words, in the United States and in Europe, the abbreviated pathway can sometimes be even more abbreviated when the above-mentioned conditions merit a waiver of confirmatory clinical trials.

Regulations in Colombia follow this trend, establishing almost the same criteria used by the FDA and EMA for waiving confirmatory human experiments. Actually, the Colombian immunogenicity guideline (4) issued in September 2016 state the following:

In the case of competitor therapeutic proteins, it is required that similarity on safety and efficacy can be clearly deduced from physicochemical characteristics, biological activity/potency and pharmacokinetic (PK) and/or pharmacodynamic (PD) profiles of the biosimilar *and* the reference product or pharmacopoeic standard (when the latter can be used for physicochemical characterization).

The Colombian Regulation (Decree 1782/2014) is innovative in the sense that it presents these possible waivers as a third separate pathway called the *abbreviated comparability pathway* (5). It is not unique, however, in the sense that this possibility already exists in other jurisdictions in cases when, as we said before, the abbreviated pathway is further expedited/abbreviated.

The decision to present the abbreviated comparability pathway as a separate, independent option was based on public health policy that took into account the political context at the time. It intends to incentivize the entry of quality competition, eliminating unnecessary technical barriers to trade, when possible. This public health perspective is not just a Colombian approach, it answers the call of the World Health Assembly to “work to ensure that the introduction of new national regulations, where appropriate, does not constitute a barrier to access to quality, safe, efficacious and affordable biotherapeutic products, including similar biotherapeutic products” (6). This resolution was also approved by the leadership of the Union of South American Nations in 2014 .

The technological prospective and the advancement of scientific knowledge suggest that circumstances when confirmatory human comparative studies will be unnecessary to demonstrate safety and efficacy are becoming more the rule than the exception; refer for instance to studies by Schellekens and Moors and by Grabowsky cited in our original article (2). As a result, the regular abbreviated pathway for biosimilars (in Colombia, the comparability pathway) will most likely be further abbreviated, becoming as such, the *abbreviated comparability pathway*, established by the Colombian Decree.

More specifically, it is important to clarify that the Colombian Decree mandates the adaptation and adoption of the WHO Guidelines (3). Such guidelines establish that a pharmacopoeia monograph, when it exists, should be taken into consideration for physicochemical characterization, though not for functional purpose, as our regulation does.

Furthermore, regarding the demonstration of high similarity between the proposed competitor and the reference product, it is an explicit requirement included by the Colombian Decree for both comparative pathways, as shown in Table 2 of our original article (2). Note that the Decree is a general regulation, so the specific analytical studies, animal studies, the clinical study, and any additional clinical testing must be reviewed case by case, based on further guidelines as mandated by the Decree. Guidelines on stability, good manufacturing practices, and immunogenicity have already been issued. Guidelines on comparability, pharmacovigilance, and risk management plans are forthcoming.

Moreover, as previously mentioned, the minimum clinical requirement in all cases and for all competitors is a PK/PD human study, one that also measures immunogenicity outcomes. This is why, the statement in the letter by Lietzan, “the third pathway in Colombia permits market entry on the basis of comparative characterization without human trials” is not true.

Lastly, it is true that the Colombian regulations allow a biosimilar to compare itself with a reference product that is not necessarily registered in Colombia, provided it has received marketing authorization via full dossier by one of the approved sanitary agencies listed in Article 8 of the Decree 1782/2014. The relevant issue in a comparator is access to all its information on quality, safety, and efficacy, and that is why the stress placed on the “approval via full dossier.” It does mean that the core of the discussion is the full access to the information instead of to the pathway of the authorization process itself.

Colombian regulations on biotherapeutic products are aligned with regulatory trends around the world and are congruent with technical and scientific evaluation principles for public health safety, quality, and efficacy. At the same time, Colombian regulations intentionally avoid redundant or unnecessary technical requirements, when possible. Though regulatory harmonization is needed in some circumstances, it should not deny the option to innovate according to a country’s needs. The Colombian decision to provide an option for further abbreviating the abbreviated pathway contributes, in turn, to the debate on access to biosimilars, not just from the perspective of lowering cost, but also reducing technical barriers to trade and accelerating market entry of competitors.
